# Development of a multiplex PCR assay for simultaneous detection of *Theileria annulata*, *Babesia bovis* and *Anaplasma marginale* in cattle

**DOI:** 10.1016/j.exppara.2012.11.005

**Published:** 2013-02

**Authors:** Huseyin B. Bilgiç, Tülin Karagenç, Martin Simuunza, Brian Shiels, Andy Tait, Hasan Eren, William Weir

**Affiliations:** a Faculty of Veterinary Medicine, Department of Parasitology, Adnan Menderes University, Işıklı Mevki, 09016, Aydın, Turkey; b Institute of Infection, Immunity and Inflammation, Henry Wellcome Building, University of Glasgow, Garscube Campus, Bearsden Road, Glasgow, G61 1QH, UK; c Department of Disease Control, School of Veterinary Medicine, University of Zambia, 32379 Lusaka, Zambia

**Keywords:** *Theileria annulata*, *Anaplasma marginale*, *Babesia bovis*, PCR, Multiplex PCR

## Abstract

Tropical theileriosis, bovine babesiosis and anaplasmosis are tick-borne protozoan diseases that impose serious constraints on the health and productivity of domestic cattle in tropical and sub-tropical regions of the world. A common feature of these diseases is that, following recovery from primary infection, animals become persistent carriers of the pathogen and continue to play a critical role in disease epidemiology, acting as reservoirs of infection. This study describes development and evaluation of multiplex and single PCR assays for simultaneous detection of *Theileria annulata*, *Babesia bovis* and *Anaplasma marginale* in cattle. Following in silico screening for candidate target genes representing each of the pathogens, an optimised multiplex PCR assay was established using three primer sets, cytob1, MAR1bB2 and bovar2A, for amplification of genomic DNA of *T. annulata*, *A. marginale* and *B. bovis* respectively. The designed primer sets were found to be species-specific, generating amplicons of 312, 265 and 166 base pairs, respectively and were deemed suitable for the development of a multiplex assay. The sensitivity of each primer pair was evaluated using serial dilutions of parasite DNA, while specificity was confirmed by testing for amplification from DNA of different stocks of each pathogen and other *Theileria*, *Babesia* and *Anaplasma* species. Additionally, DNA preparations derived from field samples were used to evaluate the utility of the single and multiplex PCRs for determination of infection status. The multiplex PCR was found to detect each pathogen species with the same level of sensitivity, irrespective of whether its DNA was amplified in isolation or together with DNA representing the other pathogens. Moreover, single and multiplex PCRs were able to detect each species with equal sensitivity in serially diluted DNA representing mixtures of *T. annulata*, *B. bovis* and *A. marginale*, and no evidence of non-specific amplification from non-target species was observed. Validation that the multiplex PCR efficiently detects single and mixed infections from field samples was demonstrated. The developed assay represents a simple and efficient diagnostic for co-detection of tropical theileriosis, bovine babesiosis and anaplasmosis, and may be a valuable tool for epidemiological studies aimed at assessing the burden of multiple infection with tick-borne pathogens and improving control of the associated diseases in endemic regions.

## Graphical abstract

**Graphical abstract:**

**Figure:**
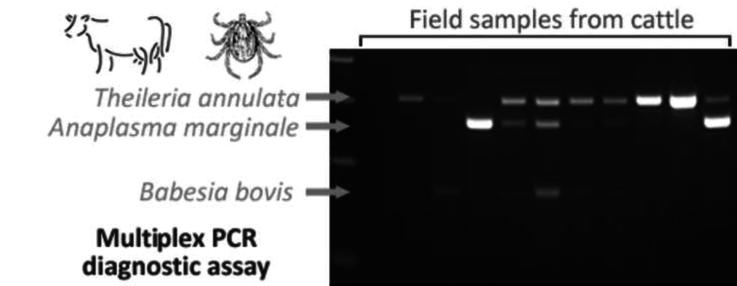

**Highlights:**

► Novel multiplex PCR for *Theileria annulata*, *Babesia bovis* and *Anaplasma marginale*. ► Specific and sensitive tool which can be applied to epidemiological studies. ► Simple and efficient assay which has been validated using field samples.

## 1 Introduction

Tick-borne diseases (TBD) pose major problems for the health and management of domestic cattle in tropical and subtropical regions of the world (Jongejan and Uilenberg, 1994). Among these diseases, tropical theileriosis, bovine babesiosis and anaplasmosis are among the most economically important (Callow 1984; Georges et al., 2001; Gubbels et al., 1999). Losses directly attributed to TBD include mortality, production losses together with the costs of veterinary diagnosis/treatment and tick control (Jonsson et al., 2008). Tropical theileriosis, caused by the protozoan parasite *Theileria annulata*, is widespread in North Africa, Southern Europe, India, the Middle East and Asia (Purnell, 1978). The disease is transmitted by several species of ixodid ticks within the genus Hyalomma (Robinson, 1982). Bovine babesiosis, caused by the protozoan species *Babesia bigemina* and *B. bovis*, is mainly transmitted by Boophilus (Rhipicephalus) ticks, although other tick species, such as *Rhipicephalus (Boophilus) evertsi*, *R. bursa*, *Ixodes ricinus* and *Ixodes persulcatus* can also transmit infection (Estrada-Pena et al., 2004; Friedhoff, 1988). Bovine babesiosis caused by *B. bovis* has been reported to be more severe than that caused by *B. bigemina* (Callow, 1984).

Bovine anaplasmosis is a tick-borne disease of cattle and wild ruminants caused by the rickettsial haemoparasite, *Anaplasma marginale*. The disease occurs in many parts of the world including North and South America, Africa, the Caribbean, Russia, European countries bordering the Mediterranean and the Middle and Far East. The disease is transmitted biologically by ixodid ticks and, in contrast to the other protozoan pathogens, may also be transmitted mechanically by biting flies or blood-contaminated fomites (Kocan et al., 2000). The distributions of *T. annulata*, *B. bovis* and *A. marginale* overlap in some geographical regions and all three pathogens are reported in a number of countries including Israel, Sudan, Egypt and China. This situation is known to occur in western Turkey where, in particular, tropical theileriosis has been shown to be prevalent (Aysul et al., 2008; Ilhan, 1999).

The pathogens that cause TBDs are often found together within a single host (Georges et al., 2001; Gubbels et al., 1999; Simuunza et al., 2011). Clinical signs, such as fever, anorexia, weight loss, reduced milk production, anaemia and icterus are commonly associated with these diseases and clinical examination of affected animals alone may not reveal the underlying pathogen species. Laboratory diagnosis in cattle is largely based on the microscopic examination of peripheral blood smears for the presence of intra-erythrocytic bodies. Parasites may be distinguished on the basis of morphological properties and this may be augmented by examining lymph node biopsy smears, particularly for the detection of *T. annulata* macroschizonts (Anon, 1997; Callow, 1984). However, a common feature of these diseases is that, following recovery from primary infection, animals become persistent carriers of the disease for an extended period of time (Callow, 1984; Neitz, 1957) and, in the case of *A. marginale*, this may be life-long (Kocan et al., 2000). It is difficult to detect infection using stained blood smears of carrier animals as the level of the pathogen is normally too low in the bloodstream (Figueroa et al., 1993). However, detection is important, as carrier animals play a critical role in epidemiology of TBD since they serve as reservoirs of infection for naive ticks and introduction of disease to new regions.

Current serological assays such as the immunofluorescent antibody test (IFAT), used for the diagnosis of Theileria, Babesia and Anaplasma, suffer from the drawback that cross-reactivity of antibodies between species can obscure test specificity and pathogen identification (Burridge et al., 1974; Kiltz et al., 1986; Kocan et al., 2000; Molad et al., 2006). Moreover, serological tests, such as the enzyme-linked immunosorbent assay (ELISA), may lack sufficient sensitivity to detect evidence of infection in samples from cattle harbouring low parasite burdens. This has important implications for disease control, since outbreaks may occur when carrier cattle, which have been incorrectly diagnosed as being clear of infection, are transported to non-endemic areas.

Although individual PCR assays designed to detect single species one at a time are effective, they can be time consuming and expensive when applied to a large number of samples that may be co-infected with a number of pathogen species. The reverse line blot (RLB) assay has overcome this problem to a large extent by allowing simultaneous detection of multiple species of pathogen in a sample (Georges et al., 2001; Gubbels et al., 1999). However, RLB requires expertise and specialised equipment and the protocol is very labour-intensive. Therefore, there is a need for a single, lower-cost and technically less demanding method that could differentially detect pathogens for diagnostic and epidemiological assessment of TBD in endemic regions. Multiplex polymerase chain reaction (mPCR) is a variant of PCR in which two or more target loci from one or more organisms are amplified using a mixture of locus-specific primer pairs in a single reaction (Edwards and Gibbs, 1994; Henegariu et al., 1997; Markoulatos et al., 2002). Thus, multiplex PCR offers a significant advantage over single-species detection systems for assessment of co-infection in a large number of samples. Indeed, mPCR assays have been shown to be valuable in field studies for the detection of viruses (Heredia et al., 1996; Markoulatos et al., 2000), bacteria (Courtney et al., 2004; Hendolin et al., 1997) and parasites, including haemoparasites and nematodes (Figueroa et al., 1993, 1996, 1998; Harris et al., 1998; Zarlenga et al., 2001). The present study describes the development of a mPCR assay for simultaneous detection of *T. annulata*, *B. bovis* and *A. marginale* in cattle-derived blood samples and evaluates its use compared to the respective single component PCR assays.

## 2 Materials and methods

### 2.1 Parasite material

A number of *Theileria*, *Babesia* and *Anaplasma* species were used for initial specificity screening of designed primers (see Supplementary data Table 1). These included a *T. parva* isolate from Kenya, an isolate of *T. orientalis* (formerly *T. sergenti*) from Japan, an isolate of *T. lestoquardi* from Iran and twelve *T. annulata* isolates: *T. annulata* (Ankara/D7) clonal cell line derived from *T. annulata* Ankara (Shiels et al., 1992) and eleven other isolates of *T. annulata* from Turkey, Tunisia, Iran, Sudan, Morocco, Israel and India. Nine *B. bovis* isolates were from countries as diverse as Mexico, South Africa, Australia, Israel and Turkey together with one isolate of *B. bigemina* from Kenya and one of *Theileria equi*. *Anaplasma* was represented by three isolates of *A. marginale* (including the St. Maries genome strain), one of *A. centrale* (Turkey) and one of *A. phagocytophila.* The material used in the study was chosen to represent a combination of isolates from a limited area and also from distant geographical areas so as to maximise the power for detecting and then excluding PCR primer pairs that showed a lack of specificity or an inability to amplify multiple isolates during the development of the mPCR assay.

A total of 73 blood samples were collected from a mixture of healthy cattle and clinical cases in the Aydın area of western Turkey where *T. annulata*, *B. bovis* and *A. marginale* are known to be endemic.

This material was used to evaluate the utility of the single and multiplex PCR protocols to amplify parasite DNA derived from bovine field samples. DNA was extracted from 100 μl blood samples using the Promega Wizard genomic DNA extraction kit (Madison, WI, USA) following the manufacturer’s instructions. Extracted DNA was re-suspended in 35 μl rehydration buffer and stored at −20 °C.

### 2.2 GenBank accession numbers and sequence resources

The GenBank accession numbers for the cytochrome b gene of different parasite species are as follows: XM949625.1 (*T. annulata*), Z23263.1 (*Theileria parva*), EU075182.1 (*Babesia bovis*) and AF109354.1 (*Babesia bigemina*). The *A. marginale* major surface protein (msp) 1β accession numbers are AY84153 and M59845. Multiple sequences were available for the *B. bovis* variant erythrocyte surface antigen–1 α subunit encoding gene and these were obtained from the Tick-borne Pathogen Genome Resources CD-ROM v1 (ICTTD–3) and the online pathogen genomic resource http://www.genedb.org.

### 2.3 Primer sequences

Putative multi-copy gene families were bioinformatically identified from available genomic databases to select suitable targets for PCR amplification of *B. bovis* and *A. marginale* DNA. In the case of *T. annulata*, the cytochrome b gene was selected and cytob1 primer set (Forward: 5′-ACT TTG GCC GTA ATG TTA AAC–3′/Reverse: 5′-CTC TGG ACC AAC TGT TTG G–3′) was used to amplify a 312 bp variable region as previously described (Bilgic et al., 2010). For the detection of *A. marginale*, the MAR1bB2 primer set (Forward: 5′-GCT CTA GCA GGT TAT GCG TC-3′/Reverse 5′-CTG CTT GGG AGA ATG CAC CT-3′) was designed to specifically amplify a 265 bp region of the major surface protein–1β encoding gene. For *B. bovis*, the bovar2A primer set (Forward: 5′-CAA GCA TAC AAC CAG GTG G–3′/Reverse: 5′-ACC CCA GGC ACA TCC AGC TA–3′) was designed to amplify a 166 bp region of the multi-copy VESA–1α gene. Each set of primer pairs was checked for specificity using the BLASTN algorithm in conjunction with the NCBI database (http://blast.ncbi.nlm.nih.gov/Blast.cgi). Primer melting temperatures (T_m_) were predicted and the potential for self-annealing, i.e. hair-pin loop and primer-dimer formation, were bioinformatically tested using oligonucleotide analyser software 1.0.2 (http://www.uku.fi/~kuulasma/OligoSoftware).

### 2.4 Single and multiplex PCR

The PCR primer sets Cytob1, MAR1bB2 and bovar2A were validated individually in order to determine their specificity. Each single PCR reaction was performed in a final volume of 50 μl containing 10 mM Tris–HCl (pH 8.3), 50 mM KCl, 1.5 mM MgCl_2_, 0.001% gelatin, 250 μM of each deoxynucleotide triphosphate (dNTP), 1 U of Ampli*Taq* DNA polymerase (Applied Biosystems, Tokyo, Japan), 10 μM of each primer and 2 μl of template DNA. The reactions were performed using an automatic thermal cycler (Techne). Reaction conditions comprised an initial denaturation step of 94 °C for 3 min followed by 30 cycles of denaturation (95 °C for 50 s), primer annealing (50 °C for 50 s) and extension (65 °C for 1 min). A final extension at 65 °C for 10 min was performed. For each reaction, 10 μl of PCR product was electrophoresed on a 1.5% agarose gel containing 10 μl/ml ethidium bromide in Tris–acetate-EDTA (TAE) buffer at 100 V and visualised under UV light.

A multiplex PCR protocol was developed based on the study by Henegariu et al. (1997). The optimised multiplex PCR was performed in a final volume of 50 μl containing 13 mM Tris–HCl (pH 8.3), 65 mM KCl, 2 mM MgCl_2_, 0.0013% gelatin, 300 μM of each dNTP, 1 U of Ampli*Taq* DNA polymerase, 0.5 μM of each cytob1 primer, 0.2 μM of each MAR1bB2 primer and 0.4 μM of each bovar2A primer and 2 μl of template DNA. Reaction conditions comprised an initial denaturation step of 94 °C for 5 min followed by 30 cycles of denaturation (95 °C for 50 s), primer annealing (56 °C for 50 s) and extension (65 °C for 50 s). A final extension at 65 °C for 5 min was performed. For each reaction, 10 μl of PCR product was analysed by gel electrophoresis on a 2% agarose gel, as described above.

### 2.5 Sensitivity of single and multiplex PCR

To determine the detection limit of the single and multiplex PCRs, equal amounts (200 ng) of DNA from *B. bovis* vaccine strain T, *A. marginale* (St. Maries) and *T. annulata* (Ankara/D7) were used to create three individual series of 10-fold dilutions using distilled water. In addition, equal quantities of DNA representing these three species were mixed and 10-fold serially diluted in distilled water to evaluate the sensitivities of the single and multiplex PCR assays to amplify from samples containing mixed DNA templates. Single and multiplex PCR was performed using the optimised conditions described above.

### 2.6 Cloning and sequencing

Products of multiplex PCR amplification were separated on a 2% agarose gel and visualised with 10 μg of ethidium bromide under UV illumination. Fragments corresponding to the predicted amplicons of 312 bp, 265 bp and 166 bp for *T. annulata*, *A. marginale* and *B. bovis*, respectively, were cut from the gel individually and purified using the QIAquick gel extraction kit (QIAGEN, Germany). Purified products were then ligated into the pCR4–TOPO plasmid vector (Invitrogen^TM^) and cloned by transformation into TOP10 *Escherichia coli* cells, according to the manufacturer’s instructions. Single picked colonies were grown overnight and plasmids purified using the QIAGEN plasmid purification kit (QIAGEN, Germany). To confirm plasmids contained the correct insert, 1 μg of purified plasmid DNA was digested with EcoR1 and electrophoresed on a 1.5% agarose gel. Between 1 and 2 μg DNA was purified from positive colonies and sequenced by a commercial sequencing service (MWG Biotech, Germany).

## 3 Results

### 3.1 Target identification and primer design

The genome of *A. marginale* (1.2 Mb) is considerably smaller than those of *T. annulata* (8.35 Mb) and *B. bovis* (8.2 Mb) and contains fewer multi-copy genes (Brayton et al., 2005). The major surface protein 1 of *A. marginale* (MSP1) is a dimer consisting of two polypeptides, MSP1a encoded by msp1α and MSP1b encoded by msp1β. msp1α is a single-copy gene which exhibits length polymorphism among different strains of the pathogen while msp1β corresponds to a multi-gene family, comprising two full-length and three partial open reading frames (Brayton et al., 2005). Both msp1α and msp1β contain domains of tandemly repeated sequence, although this is less pronounced in msp1β (Barbet and Allred, 1991). Two of the three partial msp1β genes were found to share 100% identity at the nucleotide level and consequently the MAR1bB2 primer set was designed to specifically amplify a 265 bp fragment of this sequence (see Supplementary data, Fig. 1B).

The *B. bovis* genome contains several multi-copy gene families including VESA1-encoding genes, SmORFs, variable merozoite surface antigen encoding genes, 40S ribosomal genes and ABC transporter family-encoding genes. Full-length and truncated forms of these sequences were represented in the available genomic databases. The *ves1* genes comprise the largest family in the *B. bovis* genome (Brayton et al., 2007) and encode the VESA1α and VESA1ß subunits (Al Khedery and Allred, 2006). Genomic analysis identified 119 putative *ves1* genes in *B. bovis*, namely 72 VESA1α-encoding genes, 43 VESA1ß-encoding genes and four unclassified genes (Brayton et al., 2007). Sequences of the genes encoding the 72 VESA1α were aligned to find a conserved region for PCR amplification. A 166 bp region, without any length polymorphism, was identified in 47 of the 72 VESA1α-encoding genes. The extremities of this region were reasonably well conserved and when sequences containing single nucleotide polymorphisms (SNPs) in these regions were removed, 24 sequences remained and these were used for designing the bovar2A primer set (see Supplementary data, Fig. 1C).

For *T. annulata*, the species-specific cytob1 primer set was used to amplify a 312 bp region of *cytochrome b*. This primer set has been shown previously to be capable of detecting the parasite in carrier animals in the field with a high level of sensitivity (Bilgic et al., 2010).

BLAST analysis indicated that each primer sequence was species-specific and did not possess additional local similarity to the target sequence. Primer pairs were analysed using Oligo Analyser software 1.0.2 and the cytob1 forward primer was found to have the lowest GC content of the six oligonucleotide primers (38.1%), a predicted *T*_m_ of 54 °C and possessed the highest potential for self-annealing (−6.07 kcal/mol) (see Supplementary data, Table 2). The GC content and *T*_m_ value of the cytob1 forward primer, however, was not significantly lower than the other primers and by optimising MgCl_2_ and primer concentrations in the reaction mixture, the risk of this primer failing to anneal was overcome (data not shown). The bovar2A primers were not predicted to form loop structures and for the MAR1bB2 forward primer and cytob1 reverse primer the risk was low. The cytob1 forward primer and MAR1bB2 reverse primer were calculated to have the highest potential for self-annealing at −6.07 and −3.76 kcal/mol, respectively (see Supplementary data, Table 2). None of primer sets had a hairpin structure at the 3′ end and the potential for primer-dimer formation between bovar2A forward and MAR1bB2 reverse primer was calculated to be 7.39 kcal/mol (data not shown). Issues of loop formation, self-annealing and primer-dimer formation were overcome by optimising the multiplex PCR conditions, including cycling parameters, concentrations of primers, dNTPs and MgCl_2_ and the choice of buffer used (see Section 2).

### 3.2 Specificity of primer sets with single PCR

**Fig. 1:**
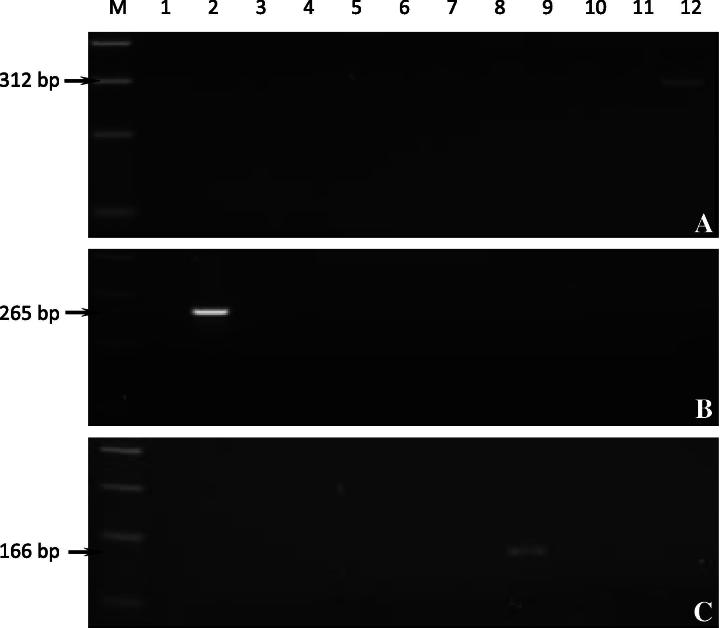
Agarose gel electrophoresis of amplified DNA from different *Theileria*, *Babesia* and *Anaplasma* species using (A) cytob1, (B) MAR1bB2 (B) and (C) bovar2A primer sets. M, 100 bp molecular size marker (Invitrogen^TM^); lane 1, negative PCR control (water); 2, *A. marginale*; 3, *A. centrale*; 4, *A. phagocytophila*; 5, *T. parva*; 6, *T. lestoquardi*; 7, *T. sergenti*; 8, *B. bigemina*; 9, *B. bovis*; 10, *T. equi*; 11, uninfected bovine DNA; 12, *T. annulata*. Arrows indicate the 312, 265 and 166 base pair amplicons specifically generated using cytob1, MAR1bB2 and bovar2A primer sets, respectively.

The specificity of cytob1, MAR1bB2 and bovar2A primer sets were investigated initially by single primer set PCR reactions (Fig. 1) using DNA from a total of twelve *Theileria*, *Babesia* and *Anaplasma* species and uninfected bovine DNA as a control. Fragments of expected size were generated from template DNA representing *T. annulata* (Aydın) (Fig. 1A, lane 12), *A. marginale* (Aydın) (Fig. 1B, lane 2) and *B. bovis* (Aydın) (Fig. 1C, lane 9) isolates using each of the species-specific primer sets, while non-target species DNA displayed no evidence of fragment amplification. This indicated that each primer set is capable of specifically amplifying the target locus in a single PCR reaction. Additionally, each primer set was tested against a number of isolates (supplementary data Table 1) of its target species and was shown to be able to generate fragments of the expected size from all isolates tested, with no evidence of length polymorphism (data not shown).

### 3.3 Sensitivity of single and multiplex PCR

**Fig. 2:**
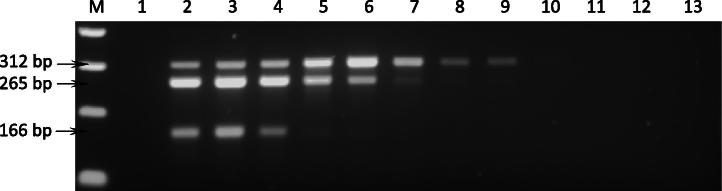
Agarose gel electrophoresis of mPCR assay of serial dilutions of a mixture of DNA from *T. annulata* (Ankara/D7), *A. marginale* (St. Maries) and *B. bovis* (vaccine strain T). M, 100 bp molecular size marker (Thermo Elect. Corp.); Lane 1, negative PCR control (water); lanes 2–12, ten-fold dilution series of mixed DNA sample ranging from 10^−1^ to 10^−11^; lane 13, uninfected bovine DNA. Arrows indicate 312, 265 and 166 base pair amplicons generated using cytob1, MAR1bB2 and bovar2A primer sets, respectively. Faint products can be observed in lane 6 for *B. bovis* (lowest band) and lane 8 for *A. marginale* (middle band).

The sensitivities of the single and multiplex PCR assays were evaluated using serially diluted DNA preparations. Starting with equal amounts of DNA from *B. bovis* vaccine strain T, *A. marginale* (St. Maries) and *T. annulata* (Ankara/D7), 10-fold serial dilutions were generated for DNA of each species individually and for a mixture representing all three species. As summarised in Table 1, the multiplex PCR was able to detect *T. annulata*, *A. marginale* and *B. bovis* at dilutions of 10^−8^, 10^−7^ and 10^−5^, with identical sensitivity for both single DNA and mixed DNA template dilutions. Agarose gel electrophoresis of mPCR amplicons from the mixed parasite DNA dilution series is shown in Fig. 2. Since identical detection limits were observed when single PCR was used to assay mixed DNA serial dilutions (Table 1) there was no loss of sensitivity using the multiplex PCR protocol compared to the single PCR protocol. When the single PCR was used to amplify DNA dilutions representing each parasite species individually, the sensitivity of the assay increased to 10^-10^, 10^−9^ and 10^−6^ for *T. annulata*, *A. marginale* and *B. bovis*, respectively (Table 1).

### 3.4 Detection of mixed infections in field samples

**Fig. 3:**
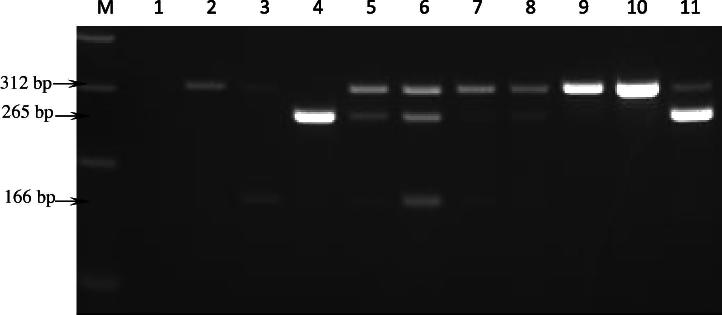
Agarose gel electrophoresis of mPCR using DNA isolated from bovine field samples. M, 100 bp molecular size marker (Invitrogen^TM^); lane 1, negative PCR control (water); lanes 2–11, template DNA isolated from bovine blood samples collected from Aydın area of Turkey endemic for *T. annulata*, *A. marginale* and *B. bovis*. Arrows indicate 312, 265 and 166 base pair amplicons generated using cytob1 (*T. annulata*), MAR1bB2 (*A. marginale*) and bovar2A (*B. bovis*) primer sets, respectively.

Seventy–three blood samples from Aydın, Turkey were examined for infection with *T. annulata*, *A. marginale* and *B. bovis*, using both the single and multiplex PCR methods. A summary of the results is shown in Table 2. Out of the 73 samples, 51, 16 and 25 samples were found to be positive for *T. annulata*, *A. marginale* and *B. bovis*, respectively, using the single PCR assay. When the samples were assayed using the multiplex PCR, 39, 1 and 17 animals were found to have single infections with *T. annulata*, *A. marginale* and *B. bovis*, respectively. A further 4, 8 and 4 animals had multiple infections with *T. annulata*/*A. marginale*/*B. bovis*, *T. annulata*/*A. marginale* and *A. marginale*/*B. bovis*, respectively. An example of the results obtained using multiplex PCR on field samples is illustrated in Fig. 3. Only minor differences were observed between the results of the single and multiplex assays with respect to co-infections. For example, two samples which were positive for both *T. annulata* and *A. marginale* by the single PCR (data not shown) showed different results for the multiplex PCR; one was positive for *T. annulata*, but negative for *A. marginale* while the other was found to be positive for *A. marginale*, but negative for *T. annulata*. In addition, multiplex PCR was able to detect a product for *A. marginale* in a sample that was negative when tested using the single PCR assay. Overall, the data from the field samples support the results of the serial DNA dilution experiment, indicating that the multiplex PCR method is capable of sensitively and specifically detecting single infections, although the multiplex method it is slightly less sensitive than the single PCR when applied to field samples.

### 3.5 Sequence analysis

The specificity of the multiplex PCR was confirmed by sequencing PCR amplicons of *T. annulata* (Turkey/D7), *A. marginale* (Turkey/Aydın) and *B. bovis* (Turkey/Aydın) strains generated using cytob1, MAR1bB2 and bovar2A primer sets, respectively. When the sequence of each amplified fragment was compared with reference sequences, the 312 bp cytob1 product showed 99% identity to cytochrome b of *T. annulata* and the 265 bp MAR1bB2 product was 100% identical to msp1β of *A. marginale* (see Supplementary data, Fig. 2A and B). Sequence derived from a single plasmid clone representing the 166 bp amplicon generated using the bovar2A primer pair showed identities ranging between 81% and 93% with the published vesa1α sequences of *B. bovis* (alignment shown in Supplementary data, Fig. 2C).

## 4 Discussion

A number of tick-borne diseases including tropical theileriosis, bovine babesiosis and anaplasmosis cause important health and management problems, resulting in reduced productivity and economic losses in domestic cattle production systems worldwide (Callow, 1984; Georges et al., 2001; Gharbi et al., 2006; Gubbels et al., 1999; Jongejan and Uilenberg, 1994). Cattle that survive acute infection with *B. bovis* and/or *A. marginale* remain persistently infected and resistant to clinical disease (Brown et al., 2006; Kocan et al., 2000). These carrier animals serve as an important reservoir of infection for ticks that go onto transmit the infection to susceptible animals (Jonsson et al., 2008; Kocan et al., 2000). A long-lasting carrier stage with low numbers of piroplasm-infected erythrocytes also occurs following recovery from acute tropical theileriosis (Neitz, 1957). Importantly, outbreaks of these diseases occur when carrier cattle are transported to non-endemic areas. Therefore, a laboratory test capable of detecting carrier-state cattle would be a useful tool to help control these diseases. Multiplex PCR, in which target sequences of different organisms can be amplified simultaneously (Edwards and Gibbs, 1994; Henegariu et al., 1997; Markoulatos et al., 2002), has been shown to be a valuable tool for the identification of various pathogen species (Figueroa et al., 1993, 1996, 1998; Harris et al., 1998; Zarlenga et al., 2001). Identification of suitable target sequences, and optimisation of reaction conditions and the specificity of each primer set are among the issues to be resolved during development of an efficient multiplex PCR assay.

It is well established that PCR performance is, to an extent, proportional to the copy number of the target sequence in the genome. Accordingly, targeting of multi-copy loci is a suitable approach in the development of sensitive PCR assays (Bilgic et al., 2010; Criado et al., 2006). In the present study, we have targeted putative multi-copy gene families in the genomes of *B. bovis* and *A. marginale* (Brayton et al., 2005, 2007; Pain et al., 2005). In the case of *T. annulata*, the *cytochrome b* locus was selected. Although this gene is single copy in the mitochondrial genome, multiple copies of the mitochondrial genome are estimated to be present in each mitochondrion (Wiesner et al., 1992). We have recently demonstrated that cytochrome b of *T. annulata* provides a very sensitive and specific gene target for the detection of carrier animals in the field (Bilgic et al., 2010). Previous studies on large multi-copy gene families in *T. annulata* (Sfi, Tar and SVSP genes) revealed that none of these gene families could provide a suitable target for amplification due to extensive nucleotide polymorphism among paralogous genes that display a high level of diversity within and between isolates (Bilgic et al., 2010; Weir et al., 2010). Analysis of the mPCR-amplified 312 bp fragment from *T. annulata* revealed that it shared 99% identity with published genomic sequences of the cytochrome b gene (see Supplementary data, Fig. 2A), while in comparison, 74% identity was shared with the orthologous sequence in *Theileria parva* (data not shown). This demonstrates that the correct target gene has been amplified and indicates a high level of conservation across *cytochrome b* sequences within the species, as anticipated (Wilson and Williamson, 1997).

Previous studies indicate that msp1β in the *A. marginale* genome is a sensitive and specific target for detection of infection both in ticks (Goff et al., 1988) and in cattle (Carelli et al., 2007; Goff et al., 1988; Molad et al., 2006). In the present study, the MAR1bB2 primer set was designed to specifically amplify a 265 bp conserved region within two msp1β genes. The primer set successfully amplified all available stocks and the sequence of PCR amplified fragments was shown to be identical at the nucleotide level to two published msp1β sequences (AY84153 and M59845) (see Supplementary data, Fig. 2B). These results suggest that the MAR1bB2 primers may be suitable to detect *A. marginale* isolates from various parts of the world. However, the sensitivity and specificity of these primers would require to be tested using a larger panel of isolates.

The ves1 genes comprise the largest multi-copy gene family in the *B. bovis* genome and mining genomic databases revealed 119 ves1 genes; 72a, 43b, and four unclassified (Brayton et al., 2007). Variant erythrocyte surface antigen 1 is a heterodimeric protein composed of alpha and beta subunits (Al Khedery and Allred, 2006) encoded by ves1α and ves1β respectively. The polypeptide subunits differ in size and are antigenically distinct (Allred et al., 1994). In this study, the bovar2A primer set was designed to specifically amplify a 166 bp region of 24 ves1α genes which is flanked with conserved priming sites. The bovar2A primer set was demonstrated to be able to amplify every member of a panel of nine geographically distinct isolates with no evidence of any length polymorphism. Sequence analyses of a single 166 bp PCR product from the *B. bovis* T vaccine strain showed 92% identity to published sequences of *B. bovis* vesa1α genes (see Supplementary data, Fig. 2C). This relatively low level of similarity may be, at least in part, due to the fact that VESA1 undergoes rapid antigenic variation in order to evade the host immune response (Allred et al., 2000; O’Connor and Allred, 2000) and may be under selective pressure to diversify. The results of the present study suggest that the bovar2A primer set may be used to detect *B. bovis* in different geographic regions where the disease is endemic. Moreover, specificity of each primer set (cytob1, MAR1bB2 and bovar2A) was confirmed with single PCR using a series of DNA samples including *Theileria*, *Babesia* and *Anaplasma* species (see Section 2). No evidence of amplification from non-target species was observed (data not shown). Based on the species-specific nature of the amplified products and the ability to reproducibly generate amplicons in mixed DNA samples, it was concluded that the primer sets in the present study were suitable for development of a multiplex PCR assay to detect the pathogens causing tropical theileriosis, bovine babesiosis and anaplasmosis. Further evidence of the assay’s specificity was revealed in the field study, when only amplicons of the expected size were generated.

The optimised multiplex PCR was able to specifically detect *T. annulata*, *B. bovis* and *A. marginale* from both single and mixed parasite DNA preparations. There was no difference in the detection limit of the multiplex PCR when using parasite DNA of either a single species or a mixture of DNA from all three species. Moreover, single and multiplex PCRs were able to detect DNA of each species with equal sensitivity in serially diluted DNA mixtures of *T. annulata*, *B. bovis* and *A. marginale* (summarised in Table 1). However, sensitivity of the single PCR when using DNA template of each species in isolation was greater than that obtained for the multiplex PCR (Table 1). It has been hypothesised that the amount of template DNA used in the reaction mixture as well as competition for a finite amount of reagents between primers affect the amount of each product generated during the reaction (Edwards and Gibbs, 1994; Henegariu et al., 1997). Thus, in the case of single PCR assays with single species DNA preparations, a high proportion of template DNA together with the lack of competition between the primers would predict that these reactions would yield the greatest amount of amplicon and that the sensitivity of the single PCR would be greater than that of the multiplex assay. Nevertheless, the multiplex PCR assay was able to amplify target genes in mixed DNA templates with the same sensitivity as the single PCR. Therefore, in terms of epidemiological studies, the multiplex PCR is, in general, as capable of detecting multiple infections as the single PCR but with the advantage of simultaneous identification of all three pathogens species in a single assay. It would be possible to enhance the sensitivity of the multiplex PCR described in the present study by incorporating a secondary detection method of the amplified products (Figueroa et al., 1993). This, however, would complicate a relatively straightforward approach and may increase the risk of contamination and subsequent generation of false positive results.

The results obtained using the field samples clearly indicate the prevalence of mixed infections in the field and verify the requirement for an assay that can simultaneously detect multiple pathogen species. Application of the multiplex PCR will facilitate the assessment of risk factors associated with TBDs, and this is important for improved design and implementation of cost-effective control strategies in endemic and non-endemic regions (Simuunza et al., 2011). Risk factors include animal movement between regions, distribution and activity of vector ticks, existence of carrier animals and the presence of multiple pathogens in individuals. To assess the latter two risk factors, the ability to detect multiple infections is essential and when integrated with data relating to the two former factors, the establishment of more effective and targeted control strategies becomes a realistic possibility.

Multiplex PCR results obtained from field samples showed that 22% of animals were co-infected with two or three parasites, while 78% were infected with only one species. Only one animal in this study was found to be negative. Only a minor discrepancy was noted in the results of the multiplex PCR compared to the single species PCR. For two samples, a single species of pathogen was detected using the mPCR while two species were detected using the single PCR. This difference was attributed to both samples having a low parasitaemia and could be due the frequency of detection decreasing with a decrease in parasitaemia (Calder et al., 1996; Martins et al., 2008).

Of the three tick-borne diseases investigated in the present study, tropical theileriosis was found to be most prevalent with 51 out of 72 parasite-infected cattle carrying *T. annulata*. Of these animals, 39 (76%) were infected only with *T. annulata* while the other 12 were co-infected with *A. marginale* and/or *B. bovis*. These results support the view that tropical theileriosis is the most prevalent tick-borne disease in Aydın region of Turkey (Aysul et al., 2008; Ilhan, 1999). While the low infection rate for *A. marginale* (17/73 = 23%) may reflect a low prevalence of the disease in the study area, seasonal fluctuations in parasitaemia (Kocan et al., 2000) may have resulted in a low level of rickettsaemia at the time of sampling. *Babesia bovis* infections in cattle are characterised by low parasitaemia during the acute phase of the disease (Schetters et al., 1998) and cattle that survive initial infection and disease-resistant calves, remain persistently infected (Brown et al., 2006; Goff et al., 2001) with very low parasite levels that are sufficient for transmitting infection to ticks. Such cattle are potentially an important factor in the maintenance of *B. bovis* in endemic areas as well as a suspect source for introduction and spread of the disease to non-endemic areas where competent vectors are present (Howell et al., 2007). These results highlight the situation in Turkey where, although infection with *T. annulata* appears to be the main risk, there is also a clear risk of co-infection with *B. bovis* and *Anaplasma*. Therefore, testing for the major pathogen species in isolation may be underestimating the overall risk, and development of assays that provide a more complete picture will be beneficial for a more complete assessment of TBD within a particular region. The multiplex assay developed in this study provides a simple test for detecting multiple tick-borne parasites in carrier animals in Turkey and should be useful for studies assessing the impact of productivity losses associated with co-infection by these pathogens.

## Figures and Tables

**Table 1:** Comparison of sensitivities of species-specific primer sets in single and multiplex PCR.

DNA dilution series	Single PCR			mPCR
	cytob1	MAR1bB2	bovar2A	
*T. annulata* alone	10^−10^	–	–	10^−8^
*A. marginale* alone	–	10^−9^	–	10^−7^
*B. bovis* alone	–	–	10^−6^	10^−5^
*T. annulata* in mixture	10^−8^	–	–	10^−8^
*A. marginale* in mixture	–	10^−7^	–	10^−7^
*B. bovis* in mixture	–	–	10^−5^	10^−5^

**Table 2:** Single and multiplex PCR tests on field bovine blood samples.

PCR reaction	Number of PCR positive animals						No. of PCR negative animals	Total no. of animals
	T.a	A.m	B.b	T.a + B.b + A.m	T.a + A.m	B.b + A.m		
cytob1	51	0	0	–	–	–	22	73
MAR1bB2	0	16	0	–	–	–	57	73
bovar2A	0	0	25	–	–	–	48	73
Multiplex PCR	39	1	17	4	8	4	1	73

T.a, B.b and A.m indicates *T. annulata*, *B. bovis* and *A. marginale*, respectively.
