# Supplementary material for: Development of a multiplex PCR assay for simultaneous detection of Theileria annulata, Babesia bovis and Anaplasma marginale in cattle
Source: Exp Parasitol. 2013 Feb;133(2):222–9. doi: 10.1016/j.exppara.2012.11.005 (PMC3650576; doi:10.1016/j.exppara.2012.11.005)
Supplement: Supplementary data 1 [file mmc1.doc]

**(A)**

**Cytob1 forward primer**

Tan ACTTTGGCCGTAATGTTAAACATTTGTTTCGGTTGGTTTGTTCGTCTTTATCACTCGTTTGGAGTTTCGTTTTATTTCTTCTTTATGTTTCTACATATCATGAAAGGTATGTGGTATTCTAGTAATCATTTACCTTGGTCTTGGTATTCTGGTGTTGTTATTTTCG

Bbov GTAATTAGAGTGCTTGCTGAAGTCAATATGGGTTGGGCAATGCGTTATTTTCACGCTCAATGTGTTTCTTTTTGTTTCTTTTTCATGATGCTACATATGCTAAAGGGATTGTGGTACTCAAGCAGATATCTACCATGGTCATGGTATTCTGGAATGGTTATATTTA

Bbig GTTATCAGAGTATTAACTGAGGTTAATATGGGTTGGGCACTTCGTTATTTCCATGCTCAATGTGTTTCTTTTTGCTTTTTCTTCATGATGTTACATATGTTAAAAGGTTTATGGTACTCAAGTAAACATTTACCTTGGTCCTGGTATTCAGGAATGGTTATATTTA

Tp CTTTCTAGACTTGTTACAGAAACACAGTTTGGTTGGTTTGTCCGTTTATATCATTCTGTTGGTGTCTCATTTTATTTCTTTTTTATGTTTATCCACATAATTAAAGGTATGTGGTATTCTAGTAAATATATGCCTTGGTCTTGGTACTCTGGCATAGTTATTTTAA

* * * * * ****** * *** * ** * * ** ** **** ** ** ** *** * * ** ** * ** ** * ***** ** ** * ** * ** ***** ***** ** ** * ***** **

**Cytob1 reverse primer**

Ta TTTTAAGTATAGCAACTGCTTTTGTTGGTTATGTATTACCAGATGGTCAAATGAGCTTCTGGGGAGCTACAGTCATAGGTGGTTTATTGAAATTTTTCGGAAAAGCTAATGTTCTAATTTTTGGAGGCCAAACAGTTGGTCCAGAG

Bbov TTTTAAGTATGGCTATTGCTTTCTTAGGTTATGTTCTTCCTTACGGACAAATGAGTTATTGGGGAGCAACAGTTATTATAAACTTGTTCTACTGGTTCCCAGATTTAGTCGCTCTTGTTTTGGGTGGTTATGGAGTTGCATTTCCA

Bbig TTTTGAGTATGGCAATTGCTTTCTTAGGTTATGTTCTTCCAAATGGACAAATGAGTTACTGGGGAGCAACAGTTATAATTAACTTATTCTATTGGTTCCCTGATATGGTATCTCTTGTACTAGGAGGATTTGGTGTTGGATTCCCA

Tp TTTTGAGTATAGTTATTGCTTTTACTGGTTATGTGTTGCCAGATGGTCAAATGAGTTTTTGGGGAGCTACAGTTATATCGAATTTGTTAGAATGGTTTGGAAAAGCCAAAGTTATAACTTTCGGTGGATTTACAGTAGGTCCAGAG

**** ***** * ******* ******** * ** * ** ******** * ******** ***** ** ** ** * * ** * * * * ** ** ** *

**(B)**

**MAR1bB2 forward primer**

AMF90 GCTCTAGCAGGTTATGCGTCAGTTGAACAGCTAGAAGAAGCAAAGGAAGCAGACAGGGTACAGGCTGAGCAGCGAGCTGAAGCACAAGCAATGACCGAGCGTGTGGCAGGGGAGCGTGCAGCAACAGTTGCTGCAGGGACTGAAACCATTAAGACCATCGTCAGCG

AMF98 GCTCTAGCAGGTTATGCGTCAGTTGAACAGCTAGAAGAAGCAAAGGAAGCAGACAGGGTACAGGCTGAGCAGCGAGCTGAAGCACAAGCAATGACCGAGCGTGTGGCAGGGGAGCGTGCAGCAACAGTTGCTGCAGGGACTGAAACCATTAAGACCATCGTCAGCG

**********************************************************************************************************************************************************************

**MAR1bB2 reverse primer**

AMF90 ATATGCGGAATGAGCTTGCTAAAGGGCATGAACAGCTTCAGCTCGTCATCACCGATATGTGTAATGAGCTTGCACAAATAGGTGCATTCTCCCAAGCAG

AMF98 ATATGCGGAATGAGCTTGCTAAAGGGCATGAACAGCTTCAGCTCGTCATCACCGATATGTGTAATGAGCTTGCACAAATAGGTGCATTCTCCCAAGCAG

***************************************************************************************************

**(C)**

**bovar2A forward primer**

**bovar2A reverse primer**

BBOV1 CAAGCATACAACCAGGTGGTCCACTACATTAGGGCTCTGTTCTACCAACTCTACTTCCTTAGGAAGCAATGTGCAGTCAAGGTCACTTACGGAGGGAAATGGCGTGAGTGTAGGTATGGGGATGGAGTAGAGTCAAAGGGGGTTATTAGCTGGATGTGCCTGGGGT

BBOV2 CAAGCATACAACCAGGTGGTACACTACATTAGGGCCCTGTTCTACCAGCTGTACTTCCTTAGGAAGCAATGTGCTGTGAAAGTGACTTGTGGAGGGAAGTGGCGTGAGTGTAGGTATGGCCAGGGGATTGTCTCTAAGGGGGTAATTAGCTGGATGTGCCTGGGGT

BBOV3 CAAGCATACAACCAGGTGGTTCACTACATTAGGGCTCTGTTCTACCAGTTGTACTTCCTTAGGAAGCAATGCGCGGTTAAAGTGACTTGTGGAGGGAAATGGCGTGAATGTAGGTATGGCCAGGGGGTAGTGTCCAAGGGGGTAATTAGCTGGATGTGCCTGGGGT

BBOV4 CAAGCATACAACCAGGTGGTACACTACATTAGGGCTCTGTTCTACCAGCTGTACTTCCTTAGGAAGCAATGTGCCGTGAAAGTTAGTTCTGGAGGCAAATGGCGTGAATGTAGGTATGGTCATGGAGTGGTGTCCAAGGGGGTAATTAGCTGGATGTGCCTGGGGT

BBOV5 CAAGCATACAACCAGGTGGTGCACTACATTAGGGCTCTGTTCTACCAACTCTATTTCCTAAGGAAACAATGTGAAGTGAAGGTTACTTGTGGAGGGAAGTGGCGTGAATGTAGGTATGGTCATGGGGTAGTTTCCAAGGGGGTAATTAGCTGGATGTGCCTGGGGT

BBOV6 CAAGCATACAACCAGGTGGTCCACTACATTAGGGCTCTGTTCTACCAGCTCTACTTCCTAAGGAAGCAATGTGCCGTGAAGGTTACTTGTGGAGGCAAATGGCGTGAATGTAGGTATGGAAATGGGGTAGTCTCCAAGGGGGTAATTAGCTGGATGTGCCTGGGGT

BBOV7 CAAGCATACAACCAGGTGGTGCACTACATTAGGGCTCTATTCTATCAACTCTATTTCCTTAGGAAGCAATGTGCCGTGAAAGTGACTTGTGGAGGCAAATGGCGTGAATGTAGGTATGGTCAGGGGGTAGTCTCTAAGGGGGTAATTAGCTGGATGTGCCTGGGGT

BBOV8 CAAGCATACAACCAGGTGGTTCACTACATTAGGGCTCTATTCTATCAACTCTATTTCCTTAGGAAGCAATGTGCGGTGAAGGTTGCTTGTGGAGGTAAATGGCGTGAGTGTAGGTATGGCAAGGATGTGGAGTCTAAGGGGGTAATTAGCTGGATGTGCCTGGGGT

BBOV9 CAAGCATACAACCAGGTGGTACACTACATTAGGGGTCTGTTCTACCAGCTCTATTTCCTTAGGAAGCAATGTGCTGTGAAAATCACTTGTGGAGGGAAGTGGAGAGAGTGTAGGTATGGTAAGGATGTGGTGTCCAAGGGGGTAATTAGCTGGATGTGCCTGGGGT

BBOV10 CAAGCATACAACCAGGTGGTCCACTACATTAGGGCTCTATTCTACCAGCTCTATTTCCTTAGGAAACAGTGTGCTGTGAAGGTAGCCCTGGGAGGGAAATGGCGTGAGTGTAGGTATGGTAAGGATGTGGTGTCCAAGGGGGTCATTAGCTGGATGTGCCTGGGGT

BBOV11 CAAGCATACAACCAGGTGGTCCACTACATTAGGGCTCTGTTCTATCAACTCTATTTTCTTAGGAAACAATGTGCCATGAAAGTGGCTCAAGGAGGCAAATGGCGTGAGTGTAGGTATGGCAAGGATGTGGTGTCCAAGGGGGTAATTAGCTGGATGTGCCTGGGGT

BBOV12 CAAGCATACAACCAGGTGGTCCACTACATTAGGGCTCTGTTCTACCAGTTGTATTTCCTTAGGAAGCAATGTGCCATGAAAGTGGCTCTAGGAGGCAAATGGCGTGAGTGTAGGTATGGGGACGGAGTAGTGTCCAAGGGGGTTATTAGCTGGATGTGCCTGGGGT

BBOV13 CAAGCATACAACCAGGTGGTCCACTACATTAGGGCTCTATTCTACCAGTTATACTTCCTTAGGAAGCAATGTGCTGTTAAAGTGGCTATGGGAGGGAAATGGCGTGAATGTAGGTATGGCAAGGATGTGGTATCCAAGGGGGTTATTAGCTGGATGTGCCTGGGGT

BBOV14 CAAGCATACAACCAGGTGGTACACTACATTAGGGCTCTATTCTATCAACTCTATTTCCTTAGGAAGCAATGTGCAGTTAAAGTGGCTCTAGGAGGGAAGTGGCGTGAATGTAGGTATGGTCATGGAGTGGTGTCCAAGGGGGTAATTAGCTGGATGTGCCTGGGGT

BBOV15 CAAGCATACAACCAGGTGGTACACTACATTAGGGCTCTGTTCTATCAACTATACTTCCTTAGGAAGCAATGTGCTGTGAAAGTCACTTGTGGAGGCAAATGGCGTGAGTGTAGGTATGGAAATGGGGTACTTGGGAAGGATGTAGTTAGCTGGATGTGCCTGGGGT

BBOV16 CAAGCATACAACCAGGTGGTACACTACATTAGGGCTCTGTTCTACCAGTTGTACTTCCTTAGGAAACAATGTGCAGTGAAAGTTACTTGTGGAGGTAAATGGCGTGAGTGTAGGTATGGTAGTGGAGTGCTTGGAAAGGATGTAGTTAGCTGGATGTGCCTGGGGT

BBOV17 CAAGCATACAACCAGGTGGTACACTACATTAGGGCTTTGTTCTACCAGTTGTACTTCCTTAGGAAGCAATGTGCAGTGAAAGTTACTTGTGGAGGTAAATGGCGTGAGTGTAGGTATGGTAGTGGAGTGCTTGGAAAGGATGTAGTTAGCTGGATGTGCCTGGGGT

BBOV18 CAAGCATACAACCAGGTGGTCCACTACATTAGGGCCCTGTTCTACCAACTCTACTTCCTTAGGAAGCAATGTGCCATGAAAGTGGCTCTAGGAGGCAAATGGCGTGAATGTAGGTATGGTGATGGGGTGCTTGGGAAGGATGTGGTTAGCTGGATGTGCCTGGGGT

BBOV19 CAAGCATACAACCAGGTGGTCCACTACATTAGGGCTCTGTTCTACCAGTTGTACTTCCTAAGGAAGCAATGTGCCATGAAAGTGGCTCTAGGAGGCAAATGGCGTGAGTGTAGGTATGGCAGTGGAGTGCTTGGGAAGGATGTAGTTAGCTGGATGTGCCTGGGGT

BBOV20 CAAGCATACAACCAGGTGGTACACTACATTAGGGCTCTATTCTACCAGTTGTACTTCCTTAGGAAGCAATGTGCAGTTAAAGTGGCTCAAGGAGGAAAATGGCGTGAGTGTAGGTATGGGGATGGAGTGCTTGGGAAGGATGTGGTTAGCTGGATGTGCCTGGGGT

BBOV21 CAAGCATACAACCAGGTGGTACACTACATTAGGGCTCTATTCTACCAACTCTATTTCCTTAGGAAGCAATGTGCAGTTAAAGTGGCTCTAGGAGGGAAATGGCGTGAATGTAGGTATGGTAAAGATGTGCTTGGGAAGAATGCCATTAGCTGGATGTGCCTGGGGT

BBOV22 CAAGCATACAACCAGGTGGTTCACTACATCAGGGCTCTATTCTACCAGTTGTACTTCCTTAGGAAGCAATGTGCAGTTAAAGTTGCTCTAGGAGGCAAATGGCGTGAATGTAGGTATGGCAGTGGCGTGCTTGGGAAGGGGGTAATTAGCTGGATGTGCCTGGGGT

BBOV23 CAAGCATACAACCAGGTGGTACACTACATTAGGGCTTTGTTCTACCAGTTGTACTTCCTTAGGAAACAGTGTGCAGTTAAAGTGGCCCTGGGAGGGAAGTGGAGAGAGTGTAGGTATGGCAGTGGAGTGCTTGGGAATGGGGTAATTAGCTGGATGTGCCTGGGGT

BBOV24 CAAGCATACAACCAGGTGGTTCACTACATTAGGGCTCTGTTCTATCAACTCTATTTCCTTAGGAAGCAATGTGCCGTGAAGGTGGCTCTAGGAGGGAAATGGCGTGAGTGTAGGTATGGTAGTGGAGTGCTTGGGAAGGGGGTAATTAGCTGGATGTGCCTGGGGT

******************** ******** **** * ***** ** * ** ** ** ***** ** ** * * ** * ***** ** *** * ** *********** * * ** * *********************

**Supplementary data Figure 1. Alignment of published parasite gene sequences showing primer loci for species-specific PCR**

(A) *Cytochrome b* of *T. annulata* (Tan), *B. bovis* (Bbov), *B. bigemina* (Bbig) and *T. parva* (Tp); (B) conserved region of *msp1α* of *A. marginale*; (C) *vesa1α* sub-unit of *B. bovis*. ‘*’ denotes conserved nucleotides.

**(A)**

**cytob1 forward primer**

**primer**

**1 166**

D7 ACTTTGGCCGTAATGTTAAACATTTGTTTCGGTTGGTTTGTTCGTCTTTATCACTCGTTTGGAGTTTCGTTTTATTTCTTCTTTATGTTTCTACATATCATGAAAGGTATGTGGTATTCTAGTAATCATTTACCTTAGTCTTGGTATTCTGGTGTTGTTATTTTCG

Tan ACTTTGGCCGTAATGTTAAACATTTGTTTCGGTTGGTTTGTTCGTCTTTATCACTCGTTTGGAGTTTCGTTTTATTTCTTCTTTATGTTTCTACATATCATGAAAGGTATGTGGTATTCTAGTAATCATTTACCTTGGTCTTGGTATTCTGGTGTTGTTATTTTCG

**************************************************************************************************************************************** *****************************

**cytob1 reverse primer**

**167 312**

D7 TTTTAAGTATAGCAACTGCTTTTGTTGGTTATGTATTACCAGATGGTCAAATGAGCTTCTGGGGAGCTACAGTCATAGGTGGTTTATTGAAATTTTTCGGAAAAGCTAATGTTCTAATTTTTGGAGGCCAAACAGTTGGTCCAGAG

Tan TTTTAAGTATAGCAACTGCTTTTGTTGGTTATGTATTACCAGATGGTCAAATGAGCTTCTGGGGAGCTACAGTCATAGGTGGTTTATTGAAATTTTTCGGAAAAGCTAATGTTCTAATTTTTGGAGGCCAAACAGTTGGTCCAGAG

**************************************************************************************************************************************************

**(B)**

**MAR1bB2 forward**

**primer**

**1 166**

Aydin GCTCTAGCAGGTTATGCGTCAGTTGAACAGCTAGAAGAAGCAAAGGCAGCAGACAGGGCACAGGCTGAGCAGCAAGCTGAAGAACAAGCAATGACCAAGAGTGTGGCACAGGAGCGTGCAGCAACAGTTGCTGCAGGGACTGAAACCATTAAGACCATCGTCAGCG

AM90 GCTCTAGCAGGTTATGCGTCAGTTGAACAGCTAGAAGAAGCAAAGGAAGCAGACAGGGTACAGGCTGAGCAGCGAGCTGAAGCACAAGCAATGACCGAGCGTGTGGCAGGGGAGCGTGCAGCAACAGTTGCTGCAGGGACTGAAACCATTAAGACCATCGTCAGCG

AM98 GCTCTAGCAGGTTATGCGTCAGTTGAACAGCTAGAAGAAGCAAAGGAAGCAGACAGGGTACAGGCTGAGCAGCGAGCTGAAGCACAAGCAATGACCGAGCGTGTGGCAGGGGAGCGTGCAGCAACAGTTGCTGCAGGGACTGAAACCATTAAGACCATCGTCAGCG

**********************************************************************************************************************************************************************

**MAR1bB2 reverse primer**

**167 265**

Aydin ATATGCGGAATGAGCTTGCTAAAGGGCATGAACAGCTTCAGCTCGTCATCACCGATATGTGTAATGAGCTTGCACAAATAGGTGCATTCTCCCAAGCAG

AM90 ATATGCGGAATGAGCTTGCTAAAGGGCATGAACAGCTTCAGCTCGTCATCACCGATATGTGTAATGAGCTTGCACAAATAGGTGCATTCTCCCAAGCAG

AM98 ATATGCGGAATGAGCTTGCTAAAGGGCATGAACAGCTTCAGCTCGTCATCACCGATATGTGTAATGAGCTTGCACAAATAGGTGCATTCTCCCAAGCAG

***************************************************************************************************

**(C)**

**bovar2A reverse primer**

**bovar2A forward**

**primer**

**1 166**

Aydin CAAGCATACAACCAGGTGGTCCACTACATTAGGGCTCTATTCTACCAGTTGTACTTCCTTAGGAAGCAATGTGCCGTGAAGGTCACTTGTGGAGGAAAATGGCGTGAATGTAGGTATGGGGATGGAGTGCTTGGGAGGGATGTAGTTAGCTGGATGTGCCTGGGGT

BBOV1 CAAGCATACAACCAGGTGGTACACTACATTAGGGCTCTGTTCTACCAGTTGTACTTCCTTAGGAAACAATGTGCAGTGAAAGTTACTTGTGGAGGTAAATGGCGTGAGTGTAGGTATGGTAGTGGAGTGCTTGGAAAGGATGTAGTTAGCTGGATGTGCCTGGGGT

BBOV2 CAAGCATACAACCAGGTAGTTCACTACATTAGGGCTCTGTTCTACCAACTATACTTCCTTAGGAAGCAATGTGCAGTTAAAGTGACTTGTGGAGGCAAATGGAGAGAGTGTAGGTATGGGGATGGAGTGCTTGGGAAGGATGTAGTTAGCTGGATGTGCCTGGGGT

BBOV3 CAAGCATACAACCAGGTGGTACACTACATTAGGGCTCTGTTCTATCAACTATACTTCCTTAGGAAGCAATGTGCTGTGAAAGTCACTTGTGGAGGCAAATGGCGTGAGTGTAGGTATGGAAATGGGGTACTTGGGAAGGATGTAGTTAGCTGGATGTGCCTGGGGT

BBOV4 CAAGCATACAACCAGGTGGTACACTACATTAGGGCTCTATTCTACCAACTCTATTTCCTTAGGAAGCAATGTGCAGTTAAAGTGGCTCTAGGAGGGAAATGGCGTGAATGTAGGTATGGTAAAGATGTGCTTGGGAAGAATGCCATTAGCTGGATGTGCCTGGGGT

BBOV5 CAAGCATACAACCAGGTGGTTCACTACATTAGGGCTCTATTCTATCAACTCTATTTCCTTAGGAAGCAATGTGCGGTGAAGGTTGCTTGTGGAGGTAAATGGCGTGAGTGTAGGTATGGCAAGGATGTGGAGTCTAAGGGGGTAATTAGCTGGATGTGCCTGGGGT

BBOV6 CAAGCATACAACCAGGTGGTCCACTACATTAGGGCTCTATTCTACCAGCTCTATTTCCTTAGGAAACAGTGTGCTGTGAAGGTAGCCCTGGGAGGGAAATGGCGTGAGTGTAGGTATGGTAAGGATGTGGTGTCCAAGGGGGTCATTAGCTGGATGTGCCTGGGGT

BBOV7 CAAGCATACAACCAGGTGGTCCACTACATTAGGGCTCTGTTCTACCAACTCTACTTCCTTAGGAAGCAATGTGCAGTCAAGGTCACTTACGGAGGGAAATGGCGTGAGTGTAGGTATGGGGATGGAGTAGAGTCAAAGGGGGTTATTAGCTGGATGTGCCTGGGGT

BBOV8 CAAGCATACAACCAGGTGGTACACTACATTAGGGCTCTGTTCTATCAGTTGTACTTTCTTAGGAAGCAATGTGCAGTGAAAGTGACTTGTGGAGGGAAGTGGCGTGAGTGTAGGTATGGTCAGGGGGTGGTATCCAAGGGGGTAATCAGCTGGATGTGCCTGGGGT

***************** ** ***************** ***** ** * ** ** ******** ** ***** ** ** ** * ***** ** *** * ** *********** * ** * * * **********************

**Supplementary data Figure 2. Alignment of novel sequence data with published parasite gene sequences**

(A) *Cytochrome b* of *T. annulata*: D7 (Ankara D7), Tan (genome strain, Ankara C9); (B) conserved region of *msp1α* of *A. marginale*: Aydin sequence from this study and published sequences AM90 and AM98; (C) *vesa1α* sub-unit of *B. bovis*: Aydin sequence from this study and eight published sequences BBOV1-8. ‘*’ denotes conserved nucleotides.

The sequences were obtained from gel-purified fragments of PCR amplicons generated using cytob1 (312 bp), MAR1bB2 (265 bp) and bovar2A (166 bp) primer sets. The sequences generated using cytob1 and MAR1bB2 primer sets were 99 and 100 % identical to *cytochrome b* of *T. annulata* and the major surface protein 1β gene of *A. marginale* respectively. The sequence generated using the bovar2A primer set was 92 % identical to *B. bovis* variant erythrocyte surface antigen-1*α* subunit gene.

**Supplementary data Table 1. Origin and nature of parasite stocks**

| **Genus and species** | **Stock origin** | | **Material** | **Reference** |
| --- | --- | --- | --- | --- |
|  | **Country** | **Location or name** |  |  |
| *Theileria annulata* | Turkey | Ankara (D7) | Culture | CTVM, Univ. of Edinburgh |
|  | Turkey | Akçaova | Culture | " " “ “ |
|  | Turkey | Dalama | Culture | " " “ “ |
|  | Turkey | Aydın | Culture | " " “ “ |
|  | Turkey | Pendik | Culture | Özkoc and Papino 1981 |
|  | Turkey | Diyarbakır | Culture | Weir *et al.* 2006 |
|  | Tunisia | JED–4 | Culture | Ben Miled *et al*. 1994 |
|  | Iran | Razi-S3 | Culture | Hooshmand-Rad and Hashemi-Fesharki 1968 |
|  | Sudan | Umbaneai | Culture | Shiels *et al*. 1986 |
|  | Morocco | Gharb | Culture | Ouhelli *et al*. 1989 |
|  | Israel | Tova | Culture | Pipano 1974 |
|  | India | Ode | Culture | Baylis *et al*. 1992 |
| *Theileria parva* | Kenya | Muguga | Culture | Oura *et al*. 2003 |
| *Theileria sergenti* | Japan | unknown | Culture | Katzer *et al*. 1998 |
| *Theileria lestoquardi* | Iran | Lahr | Culture | Kırvar *et al*. 1998 |
| *Babesia bovis* | Mexico | Mexico | Culture | Erp *et al*. 1978; Smith *et al*. 1978 |
|  | South Africa | Kuwanygwa | Culture | Taylor & McHardy 1979 |
|  | Mexico | M07 | Culture | Vega *et a*l. 1985 |
|  | Australia | Lismore | Culture | Kahl *et al*. 1982 |
|  | Israel | Galed field strain | Culture | Kimron Vet. Inst., Israel |
|  | Israel | Gonen field strain | Culture | “ “ “ “ |
|  | Israel | MH vaccine strain | Culture | “ “ “ “ |
|  | Israel | T vaccine strain | Culture | “ “ “ “ |
|  | Turkey | Aydın | Blood | This study |
| *Babesia bigemina* | Kenya | Muguga | Culture | Posnett *et al*. 1998 |
| *Babesia equi* | unknown | unknown | Culture | Unknown |
| *A. marginale* | Turkey | Aydın | Blood | This study |
|  | Unknown | Unknown | unknown | Unknown |
|  | “ | St. Maries | " | " " |
| *A. centrale* | Turkey | Aydın | Blood | This study |
| *A. phagocytophila* | unknown | unknown | Culture | Unknown |
| uninfected bovine PBM | Turkey | Aydın | Blood | This study |

**Supplementary data Table 2. Oligonucleotide primers used in mPCR**

| **Species name** | **Gene name** | **Copies per genome** | **Primer set name** | **Amplicon**  **Length (bp)** | **Sequence**  **(5'-3')** | **GC**  **content**  **(%)** | **Tm**  **(°C)** | **Self**  **annealing**  **(kcal/mol)*** | **Loop**  **formation**  **(kcal/mol)*** |
| --- | --- | --- | --- | --- | --- | --- | --- | --- | --- |
| ***B. bovis*** | *vesa1α*  (variant erythrocyte surface Ag-1) | **72** | bovar2A | 166 | F; CAAGCATACAACCAGGTGG | 52.6 | 56.7 | -1.81 | none |
|  |  |  |  |  | R; ACCCCAGGCACATCCAGCTA | 60 | 61.4 | -3.13 | none |
| ***A. marginale*** | *msp*1β  (major surface protein 1B) | 5 | MAR1bB2 | 265 | F; GCTCTAGCAGGTTATGCGTC | 55 | 59.4 | -1.88 | -1,76  5'-GCTCTAGCAGG  ||| T  3'-CTGCGTAT |
|  |  |  |  |  | R; CTGCTTGGGAGAATGCACCT | 55 | 59.4 | -3.84 | -1,76  5'-CTGCTTGGG  \|\|\| : )  3'-TCCACGTAAGA |
| ***T. annulata*** | *cytochrome b* | nd | cytob1 | 312 | F; ACTTTGGCCGTAATGTTAAAC | 38.1 | 54 | -6.07 | -0,56  5'-ACTTTGGCCGT  ||| : )  3'-CAAATTGTAA |
|  |  |  |  |  | R; CTCTGGACCAACTGTTTGG | 52.6 | 56.7 | -3.76 | -3,64  5'-CTCTGGACCAACT  \|\|\|\| )  3'-GGTTTG |

* - calculated using Oligo Analyser software 1.0.2

nd - not determined
